# Double jeopardy in inferring cognitive processes

**DOI:** 10.3389/fpsyg.2014.01130

**Published:** 2014-10-21

**Authors:** Mario Fific

**Affiliations:** Department of Psychology, Grand Valley State UniversityAllendale, MI, USA

**Keywords:** individual differences, averaging across subjects, factorial design, inferring cognitive processes, SFT

## Abstract

Inferences we make about underlying cognitive processes can be jeopardized in two ways due to problematic forms of aggregation. First, averaging across individuals is typically considered a very useful tool for removing random variability. The threat is that averaging across subjects leads to averaging across different cognitive strategies, thus harming our inferences. The second threat comes from the construction of inadequate research designs possessing a low diagnostic accuracy of cognitive processes. For that reason we introduced the systems factorial technology (SFT), which has primarily been designed to make inferences about underlying processing order (serial, parallel, coactive), stopping rule (terminating, exhaustive), and process dependency. SFT proposes that the minimal research design complexity to learn about *n* number of cognitive processes should be equal to 2^*n*^. In addition, SFT proposes that (a) each cognitive process should be controlled by a separate experimental factor, and (b) The saliency levels of all factors should be combined in a full factorial design. In the current study, the author cross combined the levels of jeopardies in a 2 × 2 analysis, leading to four different analysis conditions. The results indicate a decline in the diagnostic accuracy of inferences made about cognitive processes due to the presence of each jeopardy in isolation and when combined. The results warrant the development of more individual subject analyses and the utilization of full-factorial (SFT) experimental designs.

## Introduction

The central goal of cognitive modeling is to learn the underlying structure of mental processes, which essentially take place in a black box. Learning about cognitive mechanisms inside the box is challenging, as many mental processes are not consciously accessible. Therefore, a reverse engineering procedure has been used to learn about these cognitive processes: an input in the form of stimuli variations is carefully selected and fed to a black box, and an output in the form of response behavior is observed. Knowing a device's blueprint, a good engineer can control input, examine output, and identify the organization of the device's subsystems.

Unlike engineers, cognitive psychologists have to infer a blueprint from the input-output relationship. Take for example two proposed models of short-term-memory (STM) search. In a serial system the memory items are scanned in a sequential fashion. In a parallel system items are scanned simultaneously. To differentiate between these two models scientists have used memory load (number of memorized items 1–6) as the input and the response time (RT) as the output. In theory, the serial and parallel systems would make different predictions for the relationship between memory load and RT. A serial system (of limited capacity) would predict linearly increasing RT as a function of memory load size. A parallel system (but of unlimited capacity) would predict a flat RT as a function of memory load size. Thus, to learn the blueprint of the STM black box a scientist would use an input consisting of a varying number of items to be memorized, then would record the output response times. Then she would compare the results with the predictions of the serial and parallel systems and decide which is the most likely model supported by the results.

However, it is not quite that simple. One of the main obstacles to unveiling the content of a black box is noisy output. A novice scientist would be (unpleasantly) surprised to learn that hardly any two human response times are of a similar value, even when the exact same task is repeated. To illustrate, here are four recorded responses times belonging to a single subject who repeated the same STM task: 455, 245, 300, and 801 ms. The output response measures varied widely although the input to the black box had a fixed memory load size (one memorized item). The question is: Why would the same set of processes used to process one item show variability when repeated? One answer, is that RTs may vary so much because the cognitive processes, operating in a black box, are not deterministic and can naturally vary in their duration over time. Another source of measurement error can arise from individual subject differences. RT measures will vary across different subjects even when the same task is used. Although subjects might employ the same set of processes in a task, their responses will vary because the processes of interest may rely on cognitive components that process at different rates.

All of these random response fluctuations are known as measurement errors, in which each observation is considered a random departure of the response from the true value associated with the process of interest.

The question remains: Is it possible to remove the measurement error from the output variable? The most robust method for doing so is the averaging tool (data aggregation) on an increased sample size. Scientists of all different disciplines have used the averaging tool to calculate precise distances between stellar bodies, plot brain activity, compare smokers with non-smokers or simply to determine the longevity of a 9-volt battery. Fueled by the central limit theorem and the law of large numbers, the sample's average value converges to the true (expected) value. The averaging tool would replace the aforementioned noisy data set with a single sample mean RT value. The simplicity and effectiveness of the averaging tool has justified its widespread use in research. However, this simplicity does not guarantee that the data averaging tool is free of conditional assumptions.

When using the averaging tool to make correct inferences about the organization of cognitive processes^1^, researchers must be aware of an unfortunate double jeopardy.

### Double jeopardy

The first way correct inferences can be jeopardized is when observed data is averaged across subjects. Free of random variability, the averaged data should show the true results pertaining to the underlying processes. But before choosing to average data a scientist should be aware of the necessary conditional assumption: that all subjects use an identical set of cognitive operations^2^. The validity of the data averaging tool depends heavily on this assumption. Take for example a group of subjects who are all serial STM processors but each subject scans an item with a different processing rate (that is constant across different memory loads). The individual results would show a set of linearly increasing response times (RTs) as a function of memory load size, each with a different slope value. Such a slope value would indicate a measure of processing rate per one item in a serial system (Sternberg, 1966). When the averaging tool is used across subjects, the resulting function would also be linearly increasing with a slope value that is the average of the individual slope values. Thus, that averaged result is an unbiased indicator of the underlying processes, presumably showing the true parameter value of an item's serial processing rate, and not a value of random individual variations.

Several major cognitive theories have advocated the idea that humans use identical cognitive operations. Such theories include the conventionally adopted ideal observer approach, or the concept of a rational decision maker. However, that hypothesis is not tenable, and it is likely false. Consider the following case in which researchers aim to explore the cognitive processes engaged in the multiplication of numbers. Suppose that they randomly sampled half of the subjects from a Western Caucasian population and another half from an East Asian population. Westerners are more likely to use their known method of long multiplication; one multiplies the multiplicand by each digit of the multiplier and then adds up all the appropriately shifted results. Easterners may use the traditional Asian stick method (sometimes referred to as the Chinese or Japanese stick multiplication method), a more visual way of using drawn lines to find the result. The average of such data would describe a non-existing method for multiplication, as the average result placed the expectations between two very different cognitive strategies. Averaging across subjects could have a clearly detrimental effect on inferences about the processes of interest and would lead to false conclusions.

In the last decade many researchers have voiced concerns about the futility of the averaging tool in learning about the true values associated with specific cognitive operations (e.g., Estes, 1956; Maddox, 1999; Gallistel, 2009; Fific et al., 2010; Fitousi and Wenger, 2011; Koop and Johnson, 2011; Hills and Hertwig, 2012; Benjamin, 2013; Pachur et al., 2014). There is a rapidly increasing trend toward accounting for individual-specific cognitive operations in contrast to testing models based on universal cognitive operations. Accounting for individual differences is essential to assessing which model provides the best fit to experimental data (Broder and Schutz, 2009; Dube and Rotello, 2012; Kellen et al., 2013a,b; Turner et al., 2013). Evidence for individual differences has been reported in judgment strategies (e.g., Hilbig, 2008; Regenwetter et al., 2009), and the analyses of individual data have been called for repeatedly when investigating fast and frugal heuristics (Gigerenzer and Brighton, 2009; Marewski et al., 2010). On the other hand there are good reasons why aggregate data should be considered under some circumstances (Cohen et al., 2008; Chechile, 2009).

The second way correct inferences about underlying cognitive processes can be jeopardized occurs when researchers fail to create the appropriate input—that is—fail to create a minimally complex research design that is sufficient and necessary to obtain diagnostic response outputs. A non-diagnostic design does not permit differentiation between tested cognitive models as the models can mimic each other in the output. It logically follows then that the input (namely a research design), should be complex enough to allow for confident model differentiation in the output. But a more complex design is more expensive. Then the question becomes: What is the “price” one has to pay in the complexity of a design so that one can make correct inferences, and when do we start to see diminishing returns?

As in real life, the price of learning complex relations is sometimes underpaid. Take for example the above STM task research design used to make inferences about underlying serial/parallel STM processing. The design has only one independent variable of memory load and a dependent variable of response time. A researcher might believe that using say six memorized items in the input is the necessary and sufficient “price” to pay to learn about how six mental processes are organized. Here is the supposed bill: the sufficient and necessary price to pay to learn about the mental organization of a total of *n* cognitive processes (say six item comparisons) is a research design that has one independent variable with *n* number of levels. The price for one learned process is paid by one stimulus condition.

Unfortunately, using such a research design is likely to underestimate the true costs of diagnosing serial and parallel processing. This is because the serial and parallel cognitive models can easily mimic each other when only a memory load variable is used (Townsend, 1969, 1971, 1990; Townsend and Ashby, 1983).

Without a rigorous theory of how to define and measure the fundamental cognitive operations involved, minimal criteria for design complexity cannot be specified. In the absence of these criteria researchers will usually seek to increase the complexity of the research design. This is the case when cognitive models are tested by how well they can account for data across various tasks, that is, by seeking generalizability. In general it is advisable to challenge a cognitive model to account for as many possible findings when different inputs are manipulated. Only the model that can provide a good fit to as many different research conditions as possible is considered the most likely model, and those that fail to account for anything less than that are falsified^3^. So for example, the likely STM model should be able to account for all (various) observed effects (memory load, target serial position, stimulus modality, etc.) and should also be able to generalize easily to other conditions (e.g., Nosofsky et al., 2011). Although useful, generalizability doesn't precisely quantify the research design complexity value that is sufficient and necessary to diagnose the underlying cognitive structure of mental processes.

### The minimal criteria for the complexity of a research design

A recently proposed approach—the systems factorial technology (SFT)—sets the precise minimum required criteria for how complex a research design should be in order to be both sufficient and necessary to differentiate between several known properties of cognitive systems. The proposed SFT approach was designed to explore conditions under which the fundamental properties of mental processes, such as the order of processing (serial, parallel, coactive), stopping rule (terminating, exhaustive), process independence and capacity, could be inferred from data (e.g., Townsend and Ashby, 1983; Schweickert, 1985; Egeth and Dagenbach, 1991; Townsend and Nozawa, 1995; Schweickert et al., 2000). The SFT has been used in the context of various cognitive tasks: For perceptual processes (e.g., Townsend and Nozawa, 1995; Eidels et al., 2008; Fific et al., 2008a; Johnson et al., 2010; Yang, 2011; Yang et al., 2013), for visual and memory search tasks (e.g., Egeth and Dagenbach, 1991; Wenger and Townsend, 2001, 2006; Townsend and Fific, 2004; Fific et al., 2008b; Sung, 2008), for face perception tasks (Ingvalson and Wenger, 2005; Fific and Townsend, 2010), and for classification and categorization (e.g., Fific et al., 2010; Little et al., 2011, 2013).

To correctly diagnose an *n* number of cognitive processes, of an unknown cognitive system that is organized with respect to processing order, stopping rule and process dependency, SFT prescribes the following minimal criteria for a research design's complexity:

- The number independent variables used should be equal to the number of processes under examination, *n*.
- Each independent variable should vary between (at least) binary values of saliency. The saliency is operationally defined as a manipulation that selectively influences a single process of interest, such that the process is speeded up (H = high saliency) or slowed down (L = low saliency).
- The levels of all independent variables should be factorially combined, that is, orthogonally crossed with all other levels of the other variables. Thus, the total number of experimental conditions is equal to 2^*n*^.

So, if a cognitive system under investigation consists of two processes that could be organized in either a serial or a parallel fashion, then the design should include two independent variables with two levels each, factorially combined, resulting in 2^2^ = 4 conditions. If a cognitive system consists of four processes, the design should include four factors, factorially combined with at least two levels of each factor, thus resulting in 2^4^ = 32 experimental conditions.

The required research design's complexity increases exponentially with research aspirations. In practice as the number of conditions increases this means that the SFT minimal criteria for differentiating between cognitive models could require lots of conditions and trials. So it is quite understandable that researchers usually use generalizability as criteria for model testing instead. The truth is that many of these research designs do not meet the minimal SFT criteria for testing different cognitive models, leading to conclusions that could be flawed.

In studies of the optimal research design, the SFT approach utilizes a so-called full-factorial design enabling a detailed processing structure analysis. If only a fraction of the full factorial design is used then this is broadly defined as a fractional-factorial design (FFD). In general FFD designs are useful as they can provide some important insights about the processes under consideration while saving on the complexity of a research design and thus saving time and effort. However, they may fail to identify important interactions between factors. As will be detailed in the next section, it is exactly the interaction information that provides the critical insights necessary to differentiate between cognitive processes. Although there is a great deal of published research about cognitive properties that can't be characterized as utilizing the FFD research design (e.g., Sternberg, 1966; Bradshaw and Wallace, 1971; Lachmann and van Leeuwen, 2004) this study will not analyze it in detail. For simplicity sake, this paper will refer to any incomplete SFT full-factorial design as an FFD design.

The second way correct inferences can be jeopardized is when using an FFD research design a researcher acts *as if* he/she has reduced the dimensionality of a full-factorial design. As such the important critical information about how to differentiate between cognitive systems is lost. So for example, the full-factorial SFT design prescribes six variables and 2^6^ = 64 conditions to learn about six STM processes. Such could be a design in which each memory item's saliency (high-low) is factorially combined with all other memory items' saliencies (for *n* = 2 see Townsend and Fific, 2004; for up to *n* = 4 see Yang et al., 2014). If instead a researcher collapses the load variable across saliency, then the resulting design is a FFD design having only the memory load variable in the input. By collapsing across the input variables the critical test conditions are dropped out, and the minimal SFT diagnostic criteria have not been reached. Thus, the likelihood of making correct inferences about any underlying cognitive processes decreases dramatically.

The remainder of this paper will outline the basic SFT tools applied on cognitive systems with two processes. Then the author will proceed with the empirical evidence showing how SFT combined with individual subject analysis can be used to improve inferences rendered unreliable by the two jeopardies.

### A generic cognitive task

Take for example a generic short-term memory/visual memory search task: the search set consists of two items (*n* = 2) and the task is to decide whether a target item was in the search set. For simplicity the author limits the analysis to target-absent trials only, in which a subject has to search an entire search set. This is the case of an exhaustive search. The question is whether processing is serial, parallel, coactive, or none of the above. In general, limiting the analysis only on target-absent responses potentially can harm diagnostic accuracy as it neglects a possible decision criteria trade-off between target-present and target absent responses. The analysis of target-absent responses only would still be sufficient for the current illustration purposes.

### The SFT full-factorial design

The adequate minimal SFT research design of the above task should include two factors with at least two levels, thus the total number of conditions should be 2^2^ = 4.

The first factor is operationally defined as the saliency of the first item in the search set, and the second factor is defined as the saliency of the second item in the search set. The saliency has binary values which allow for speeding up or slowing down of a particular process. (In what follows, H indicates a fast process, or high item-to target dissimilarity, and L a slow process, or low item-to-target dissimilarity). The idea here is that the memorized item with high saliency is processed faster than the item with low saliency, as the H item is more dissimilar to the target. In the generic task described above the cognitive operation of item scanning requires less processing time to determine that an H item is not a target, and can reject it quicker than an L item.

**Figure 1:**
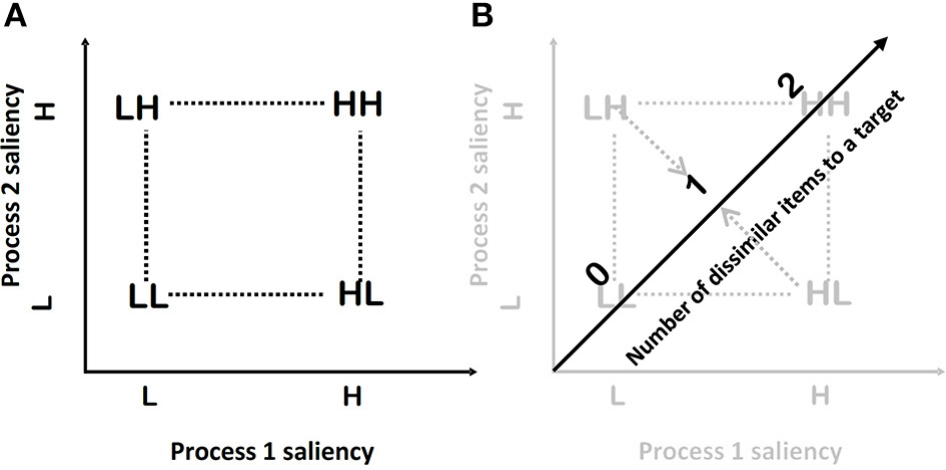
**(A)** A schematic representation of the full-factorial design. **(B)** A schematic representation of the FFD, which is obtained by collapsing the full-factorial design to a one-dimensional design across the item position factors.

In each trial two items make a search set, and thus the factorial combination of items' saliencies will result in four experimental conditions: HH, HL, LH, and LL—the so-called double factorial design (2 × 2, as employed in an analysis of variance). For example, HLindicates a condition where the first factor (processing the first item) is of high saliency and the second factor (processing of the second item) is of low saliency (see Figure 1A).

It is important to note that using the double factorial design, the different cognitive processing orders will exhibit different data patterns of mean reaction times, which brings us to the main statistical tests used in SFT.

**Figure 2:**
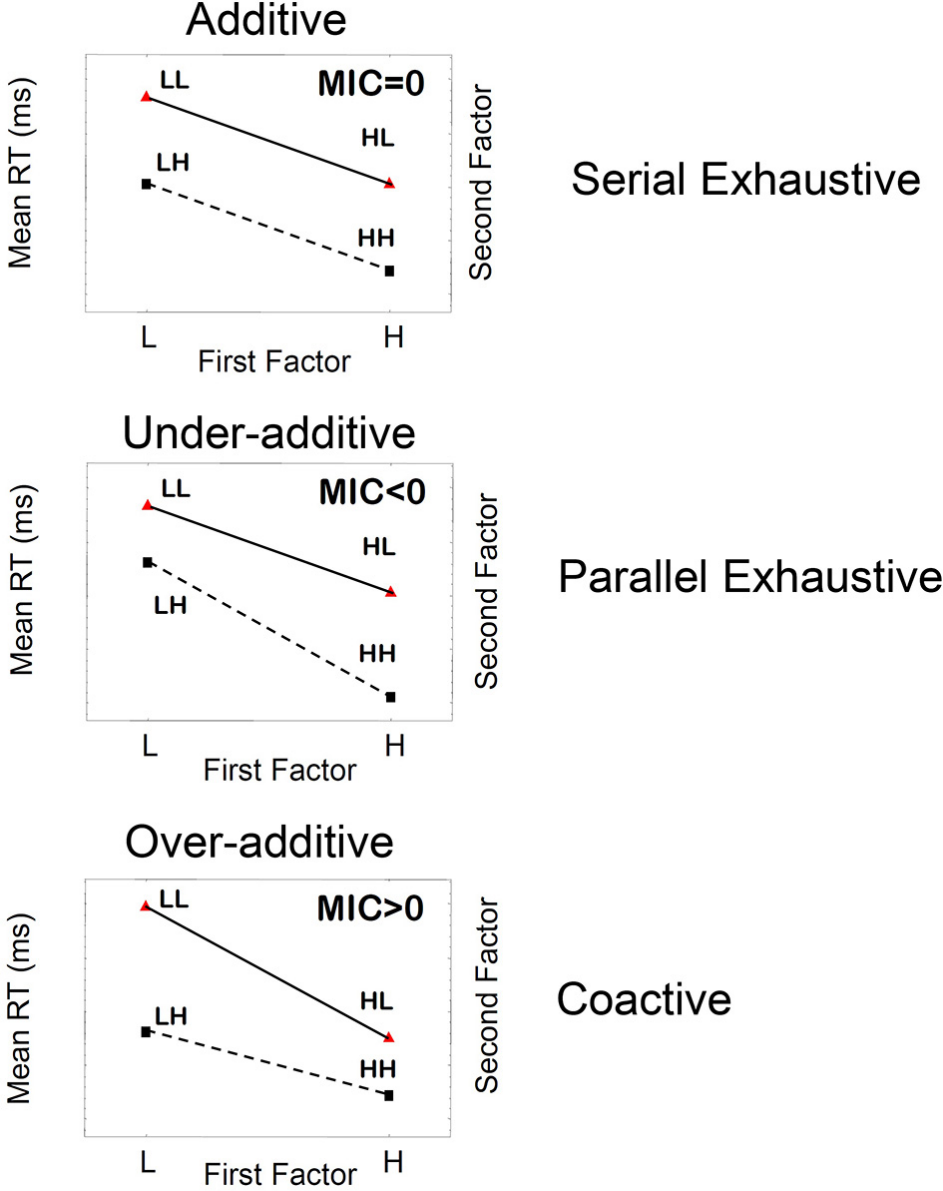
**Schematic illustration of three main patterns of mean RTs, mean interaction contrasts (MICs), and the corresponding underlying cognitive processes when the stopping rule is fixed to be exhaustive**.

Mean Interaction Contrast (MIC): The MIC statistic calculates the interaction between the factors, similarly as in an interactive analysis of variance (ANOVA) (Sternberg, 1969; see also Schweickert, 1978; Schweickert and Townsend, 1989):

(1) $$\begin{array}{l} \text{MIC}=({\text{RT}}_{\text{LL}}-{\text{RT}}_{\text{LH}})-({\text{RT}}_{\text{HL}}-{\text{RT}}_{\text{HH}})={\text{RT}}_{\text{LL}}\\ \;-{\text{RT}}_{\text{LH}}-{\text{RT}}_{\text{HL}}+{\text{RT}}_{\text{HH}} \end{array}$$

where RT is response time. This statistic is obtained by taking the double difference of mean RTs associated with each level of separate experimental factors (in this case, 2 × 2 factorial conditions). So, for example, mean RT_HL_ indicates mean response time for the condition where the first factor (processing the first item) is of high saliency and the second factor (processing the second item) is of low saliency. Figure 2 shows typical patterns of MIC tests that are expected for different processing orders, for the fixed exhaustive stopping rule.

MIC is considered a valid test providing that the following conditional assumptions hold: (a) Processing rate for any position L is always slower than H, (b) The single factors selectively influence only single sub-processes (position one and two), and (c) The independence between processes hold. Violation of any or all assumptions leads to a violation of the mean RT orderings of the experimental situations RT_LL_ > RT_LH_, RT_HL_ > RT_HH_, which is considered a quick test of the conditional assumptions.

The pattern of “additivity” is reflected by an MIC value of 0 (Figure 2). In an ANOVA, additivity is indicated by an absence of interaction between factors, thus implying that the effects of individual factors simply “add” together. This finding supports serial processing, in which the total response time is the sum of individual times stemming from each factor. Likewise, “overadditivity” is reflected by an MIC > 0 (a positive MIC), and “underadditivity” is reflected by an MIC < 0 (a negative MIC). Formal proofs of the results expressed below are provided by Townsend (1984), Townsend and Nozawa (1995) for parallel and serial systems, and for a wide variety of stochastic mental networks by Schweickert and Townsend (1989). Townsend and Thomas (1994, also see Dzhafarov et al., 2004) showed the consequences of the failure of selective influence when channels (items, features, etc.) are correlated.

If processing is strictly serial, then the MIC value will equal zero; that is, the pattern of mean RTs will show additivity. For instance, if processing is serial exhaustive, then the increase in mean RTs for LL trials relative to HH trials will simply be the result of the two individual processes slowing down, giving us the pattern of additivity illustrated in Figure 2, top panel. Parallel exhaustive processing results in a mean RT pattern of underadditivity (MIC < 0) (Figure 2, middle panel). Finally, coactive processing will lead to a pattern of overadditivity of the mean RTs (MIC > 0), as illustrated in Figure 2 bottom panel. Coactive processing is a form of parallel processing in which information from parallel processing units are pooled together into one unit, by the virtue of summation of signals from the two units. Coactivation gives rise to perceptual unitization, forming perceptual objects whose features are not analytically separable.

The SFT provides strong grounds for model comparison and model falsification, in both the non-parametric and parametric treatments of the theoretical processes. Useful statistical tools are described in several publications and are available online (Townsend et al., 2007; Houpt et al., 2014).

### The fractional-factorial design (FFD)

To get an FFD the author reduces the dimensionality of the above full-factorial design (Figures 1A,B). The resulting FFD design uses only 3 conditions from the original full-factorial design. The collapse of the full factorial design across the item position factors could be visualized as a projection of the conditions to a new single dimension (Figure 1B). I define this dimension as the number of items in a search set that are dissimilar to the target. In the HH condition, both items are dissimilar. Thus, the value is two. In the HL and LH conditions, only one item is dissimilar thus the value is one; and in the LL condition both items are similar, and thus the number of dissimilar items is zero. The observed mean RT can be plotted as a function of the number of dissimilar items, defining the RT-dissimilarity function.

Surprisingly this particular FFD design has been used in several studies to explore cognitive processes. The RT-dissimilarity function has been employed previously in the same-different judgment task (Nickerson, 1965, 1969; Egeth, 1966; Miller, 1978; Proctor, 1981; Farell, 1985; see Sternberg, 1998 for review). The general finding was that RT decreased as a number of differing dimensions between the items (Goldstone and Medin, 1994), number of dissimilar items in search set, or as a function of the structural complexity (Checkosky and Whitlock, 1973; Schmidt and Ackermann, 1990; Lachmann and van Leeuwen, 2004).

The important diagnostic feature here is the shape of the RT-dissimilarity function: if the function is strictly linear it indicates serial processing (Egeth, 1966; Posner and Mitchell, 1967; Lachmann and van Leeuwen, 2004), and if the function is non-linear it indicates parallel processing (Posner, 1978). The property of linearity can be assessed by conducting a linear regression analysis and would be shown in the coefficient of determination *R*^2^-value (e.g., Lachmann and Geissler, 2002; Lachmann and van Leeuwen, 2004, p. 11, inferred serial processing by showing linear functions, 0.98 ≤ R^2^ ≤ 0.99).

Indeed different cognitive models predict the characteristic change in RT-dissimilarity function shape. Serial exhaustive models predict that the mean RT would linearly decline as a function of item-to-target dissimilarity. Provided that a low-dissimilar item is processed slower than a high-dissimilar item, and that processing is conducted in the item-to-item fashion, the mean RT should decline with the same rate as the number of dissimilar items increases in the search set. Parallel exhaustive models predict a convex non-linear RT-dissimilarity function. In contrast, the coactive model predicts a concave non-linear RT as a function of target-to-item dissimilarity (see Supplementary Material for the derivations).

It is important to note that even though the mean RT-dissimilarity function is FFD, some diagnostic cues enable differentiation between cognitive processing strategies.

The robustness of the SFT and FFD designs to the first jeopardy: Averaging across subjects' mixed cognitive strategies and predictions of the two designs.

Neither of the two approaches is immune to the first jeopardy. When we average results of subjects who used different cognitive strategies, the resulting MIC signature and RT-dissimilarity function could reveal the most dominant cognitive system or could indicate a ghost cognitive system—a non-existing one.

Consider the generic task in which the stopping rule was set to be exhaustive. In order to make a correct decision all memorized items in the search set have to be processed. Each cognitive strategy (serial, parallel, and coactive) could be used to search the search set, but some strategies may be more preferable under certain conditions. Serial processing could be employed when it is advantageous to invest all attention to one unit at a time with a possibility for early termination. Parallel processing may be employed when all information is available and the cognitive system does not see possible limitations due to capacity sharing between multiple concurrently processed items. Coactive processing may be involved with processes that have historically occurred together and thus built a joint path in the cognitive system (perhaps a neural unit). More importantly, what is unknown to researchers is whether or not each of these cognitive processing strategies may be individual subject specific. It could be expected that some human subjects have developed more reliance on some of these strategies than on the others.

In the SFT design the following three MIC signatures could be observed. Subjects could either exhibit a parallel search, showing the underadditive MIC pattern (Figure 2 middle), a serial search showing the additive MIC (Figure 2, top), or a coactive search (parallel but not independent processes) showing the overadditive MIC pattern (Figure 2 bottom). Provided that the base rate for each processing strategy is the same, the results of averaging across subjects would predict convergence to the MIC additive signature.

Similarly in the FFD design, the subjects would show all three types of curving in the RT-dissimilarity function, concave, convex and linear. The average outcome RT-dissimilarity function would tend to converge to the linear function.

A surprising result will occur when sampled subjects are only parallel and coactive processors: a ghost cognitive strategy will be inferred. Both the averaged MIC and the RT-dissimilarity would indicate serial processing (additive MIC and linear RT function), despite that not a single subject could be characterized as such.

### The comparison test

The main goal of the current paper is to explore how effective the mean RT analysis methods are in inferring the organization of cognitive processes when both jeopardies are in place. Thus, this study cross combined the two jeopardies and compared the four resulting conditions (Table 1).

**Table 1:** **Cross combination of the levels of the two jeopardies in a 2 × 2 analysis, leading to four different analysis conditions**.

	**Analysis level**	
	**Individual**	**Group**
**RESEARCH DESIGN**		
Full factorial (MIC)	0	1
Fractional factorial (regression)	2	3

*The first jeopardy is defined as the difference between the individual and group subject analyses with regard to inferring the details associated with the cognitive processes of interest. The second jeopardy is defined as the difference between the full- and fractional-research designs with regard to inferring those same details*.

As a reference point the author will analyze the data from Condition 0 which both adheres to the SFT minimal criteria for the correct diagnosing of cognitive processes, and is based on individual subjects analyses (Table 1). Condition 0 uses the previously published MIC results of individual subject data on a large number of trials possessing lots of statistical power (Townsend and Fific, 2004; Fific et al., 2008b).

In Condition 1, the author tests the effect of the across-subject averaging on MIC test accuracy in identifying cognitive processes. In Condition 2 the author tests the effect of using an FFD design on making inferences regarding the individual subjects' data, using a regression analysis of the RT-dissimilarity function. Finally, in Condition 3 the data will be exposed to both jeopardies: the averaging across subjects and the design marginalization using FFD. In this condition the author analyzes the group mean RT-dissimilarity functions using linear regression analysis.

The expectation is that when compared to Condition 0 the three conditions will show deterioration in their ability to correctly diagnose cognitive processes. Most of the misdiagnoses should be observed in Condition 3. Although the current expectations could be logically derived from earlier works, such systematic evidence is sparse. The author hopes that the current study will illuminate both the role of individual subject analysis and the application of SFT in learning about cognitive processes.

## Methods

The results reported in this section are based on the reanalysis of data collected in previous studies (Townsend and Fific, 2004; Fific et al., 2008b). Specific details about the participants and stimuli are presented in the original papers. Here I outline the details which are pertinent to the current investigation.

### Participants

Five participants, 2 females and 3 males participated in a short-term memory search study (Townsend and Fific, 2004). Four participants, two females, and two males participated in a visual search study (Fific et al., 2008b); four participants, three females, and one male participated in the visual search study on patterns (Fific et al., 2008b). All participants were paid for their participation.

### Stimuli

#### Short-term memory study (Townsend and Fific, 2004)

Stimuli were pseudo-words in consonant-vowel-consonant (CVC) form. Two items made a search set, presented on different search-set positions (first, second). To produce the saliency effect, we manipulated phonemic dissimilarity of a search set-item to the target item. The items were drawn from two sets of phonologically confusable Serbian language consonants: fricatives (F, S, V) and semi-vocals (L, M, N). We generated different dissimilarity of search-set items to the target item by constructing the target and test items from letters drawn either from the same group or from different groups.

#### Visual search on pseudowords (experiment 1, Fific et al., 2008b)

Stimuli were Cyrillic letter-strings constructed from letters of the Serbian alphabet. The visual complexity of the letter-string stimuli was manipulated by varying the number of letters that made up a single item (1, 2, or 3 consonants). The saliency effect was produced by manipulating the degree of visual dissimilarity between the item and the target items. We employed two sets of letters: letters with curved features and letters with straight-line features. We generated different dissimilarity of search-set items to the target item using the same principles as in the above study.

#### Visual search on visual patterns (experiment 2: Fific et al., 2008b)

As stimuli, we used meaningless visual patterns taken from Microsoft's Windows standard fonts.

### Design and procedure

#### Short-term memory search (Townsend and Fific, 2004)

Each trial consisted of a fixation point and warning low-pitch tone for 1 s, successive presentation of two items in the search set for 1200 ms, an inter-stimuli interval (ISI), and a target. The ISI was defined as the interval between the offset of a search set and the onset of the target. The ISI period started with a fixation point and a second warning high-pitch tone which lasted for 700 ms. Onset of this second warning signal was activated so that its end coincided with the end of the ISI period.

The task was to decide whether a target was presented in a search set. The target was randomly chosen to be present in one-half of the memory set trials and absent in the other half Participants signified their answer, “yes” with one index finger and “no” with the other. Only target-absent trials were analyzed.

The analyzed research design consisted of the three within-subject factors: Inter-stimulus interval (ISI, 700 and 2000 ms) × Dissimilarity of item in position one (H,L) × Dissimilarity of item in position two (H, L). The last two factors constituted the full factorial SFT design permitting the assessment of processing order.

Participants ran around 44 blocks of 128 trials each. Each block was divided into 6 sub-blocks of 20 trials (except the last one which had 28 trials). The participants were requested to achieve very high accuracy, and usually only one block was completed on a particular test day. Thus, each mean RT in a specific ISI condition and particular factorial combination possessed between 300 and 400 trials per participant (depending on duration of participation). Brief rest periods were allowed every 24 trials.

The ISI was manipulated between blocks, whereas factorial combinations (HH, HL, LH, LL) varied within blocks.

#### Visual search on pseudowords (experiment 1, Fific et al., 2008b)

Each trial started with a fixation point that appeared for 700 ms and a low-pitch warning tone of 1000 ms, followed by the presentation of the target item for 400 ms. Then, a mask was presented for 130 ms, followed by two crosshairs that indicated the positions of the two upcoming test items that made the search set. A high pitch warning tone was then played for 700 ms, followed by the presentation of the two items in the search set.

The task was to decide whether or not the target was presented in the search set. Half of the trials were target present and half were target absent. On each trial, the participant had to indicate whether or not the target item appeared on the search set by pressing either the left or the right mouse key with his or her corresponding index finger. RTs were recorded from the onset of the test display, up to the time of the response. Participants were asked to respond both quickly and accurately. Only target-absent trials were analyzed.

The analyzed research design consisted of three within-subject factors: Stimulus complexity (C = 1, 2, or 3) × Dissimilarity of item in the left position (H, L) × Dissimilarity of item in right position (H, L). The stimulus complexity was operationally defined as the number of letters used to form the stimulus items. The last two factors constituted the full factorial SFT design permitting the assessment of processing order.

The two test items in the most complex condition (C = 3, with the widest stimuli) spanned 5 cm horizontally. At a viewing distance of 1.7 m from the computer screen, this width corresponds to a visual angle of 1.86 degrees, well within the fovea.

Each participant performed on 30 blocks of 128 trials each. The order of trials was randomized within blocks. The complexity of the presented items (i.e., the number of letters: C = 1, 2, or 3) was manipulated between blocks, whereas factorial combinations (HH, HL, LH, LL) varied within blocks. For each participant, the mean RT for each conjunction of item complexity and factorial combination was calculated from approximately 200 trials.

#### Visual search on visual patterns (experiment 2, Fific et al., 2008b)

This condition was identical to the C = 1 condition of the previous study, except that it employed visual patterns as stimuli instead of letters. Each participant performed in 10 blocks of 128 trials.

## Results

### Condition 0: individual subject data, MIC analysis

The results of the MIC tests are published elsewhere (Townsend and Fific, 2004; Fific et al., 2008b). The author summarizes the findings in Table 2.

**Table 2:** **Summarized ANOVA results for the MIC tests at different levels of subject analysis**.

	**MIC test ANOVA**			**Full-factorial design conditions**					
	**df 2**	***F***	**η^2^**	**LL (ms)**	**LH (ms)**	**HL (ms)**	**HH (ms)**	***MIC* (ms)**	**Inference**
**GRAND MEAN**									
	23992	15.4	0.001	772	662	662	572	20	Coactive
**VISUAL SEARCH: PSEUDOWORDS**									
*Mean subjects*	7458	50.2^**^	0.007	984	730	762	587	78	Coactive
**Complexity**									
C = 1	595	3.1^†^	0.005	619	564	623	530	−38	–
C = 2	633	34.9^**^	0.052	1106	711	802	581	175	Coactive
C = 3	631	4.4^*^	0.007	1302	885	934	579	63	Coactive
C = 1	591	1.2	0.002	557	506	509	470	12	Serial
C = 2	630	41.0^**^	0.061	908	649	681	554	132	Coactive
C = 3	626	3.1^†^	0.005	1149	799	848	549	51	Serial
C = 1	590	0.9	0.001	622	577	561	534	19	Serial
C = 2	632	59.4^**^	0.086	963	671	643	527	176	Coactive
C = 3	628	14.0^**^	0.022	1191	856	808	578	106	Coactive
C = 1	595	2.0	0.003	678	639	631	609	17	Serial
C = 2	633	33.8^**^	0.051	1194	869	949	766	142	Coactive
C = 3	630	11.7^**^	0.018	1446	995	1113	753	91	Coactive
**VISUAL SEARCH: PATTERNS**									
*Mean subjects*	2346	5.4^*^	0.002	668	577	587	532	36	Coactive
**Complexity**									
C = 1	587	2.1	0.004	863	699	746	630	49	Serial
C = 1	576	4.7^*^	0.008	750	642	655	617	70	Coactive
C = 1	584	2.0	0.003	520	469	465	432	17	Serial
C = 1	587	4.4^*^	0.007	545	494	482	452	22	Coactive
**SHORT-TERM MEMORY SEARCH**									
*Mean subjects*	14180	11.0^**^	0.001	676	641	622	571	−15	Parallel
**Interstimulus interval**									
ISI = 700	1375	0.8	0.001	606	565	559	507	−12	Serial
ISI = 700	1645	0.2	0.000	632	607	595	565	−5	Serial
ISI = 700	1202	11.4^**^	0.009	598	590	562	518	−37	Parallel
ISI = 700	1394	2.2	0.002	747	706	690	664	15	Serial
ISI = 700	1439	0.3	0.000	786	703	666	593	10	Serial
ISI = 2000	1379	0.3	0.000	628	567	561	507	7	Serial
ISI = 2000	1710	3.7^†^	0.002	640	628	600	567	−21	Serial
ISI = 2000	1201	14.7^**^	0.012	613	592	577	512	−43	Parallel
ISI = 2000	1387	5.6^*^	0.004	748	730	717	672	−27	Parallel
ISI = 2000	1412	4.2^*^	0.003	761	708	680	591	−36	Parallel

** *p < 0.01*,

* *p < 0.05*,

† *p < 0.08. The df1s were 1*.

All subjects' results satisfied the ordering of mean RTs (RT_LL_ > RT_LH_, RT_HL_ > RT_HH_), except for the first subject in the C = 1 condition of the visual search task (Table 2). In addition, all subjects showed significant main effects of the single factors, that is, the effect of high and low dissimilarity for each item position. Highly dissimilar items always showed on average faster processing rates than the low dissimilar items, for both item positions (1 and 2). These findings indicated that the basic manipulation of item-to-target dissimilarity produced the expected cognitive effect and furthermore that the processing of an item in each particular position occurred. Being uniform for all subjects, these results were not reported in the table.

The critical MIC test results were based on the inspection of the significance of an interactive component and the sign value of the MIC score. As reported in Table 2 the individual-subject analyses showed individual subject variability in MIC values. All MIC values were interpretable (except the first subject in the C = 1 condition), and the signatures each fell into one of the expected categories.

#### Conclusion

The subjects' MIC values showed large variability across the three experiments. In the two visual studies subjects showed primarily over-additive results (9 subjects) and some additive results (6 subjects), thus implying coactive and serial processing. One subject's results were inconclusive, violating the conditional assumptions of selective influence and or process independence. The subject could also exhibit an unknown type of cognitive strategy. In contrast, the subjects in the memory study showed either additivity (6 subjects) or under-additivity (4 subjects), thus implying the presence of both serial and parallel processing across subjects. See Table 3 for summary.

**Table 3:** **Summary of the inferences across different comparison conditions from Table 1**.

	**Serial**	**Parallel**	**Coactive**	**Unaccounted**
Condition 0 (full)	12	4	9	1
Condition 1 (jeop 1)	–	10	16	–
Condition 2 (jeop 2)	13	4	9	–
Condition 3 (jeop 1 and 2)	26	–	–	–

### Condition 1: averaged subjects data, MIC analysis

**Figure 3:**
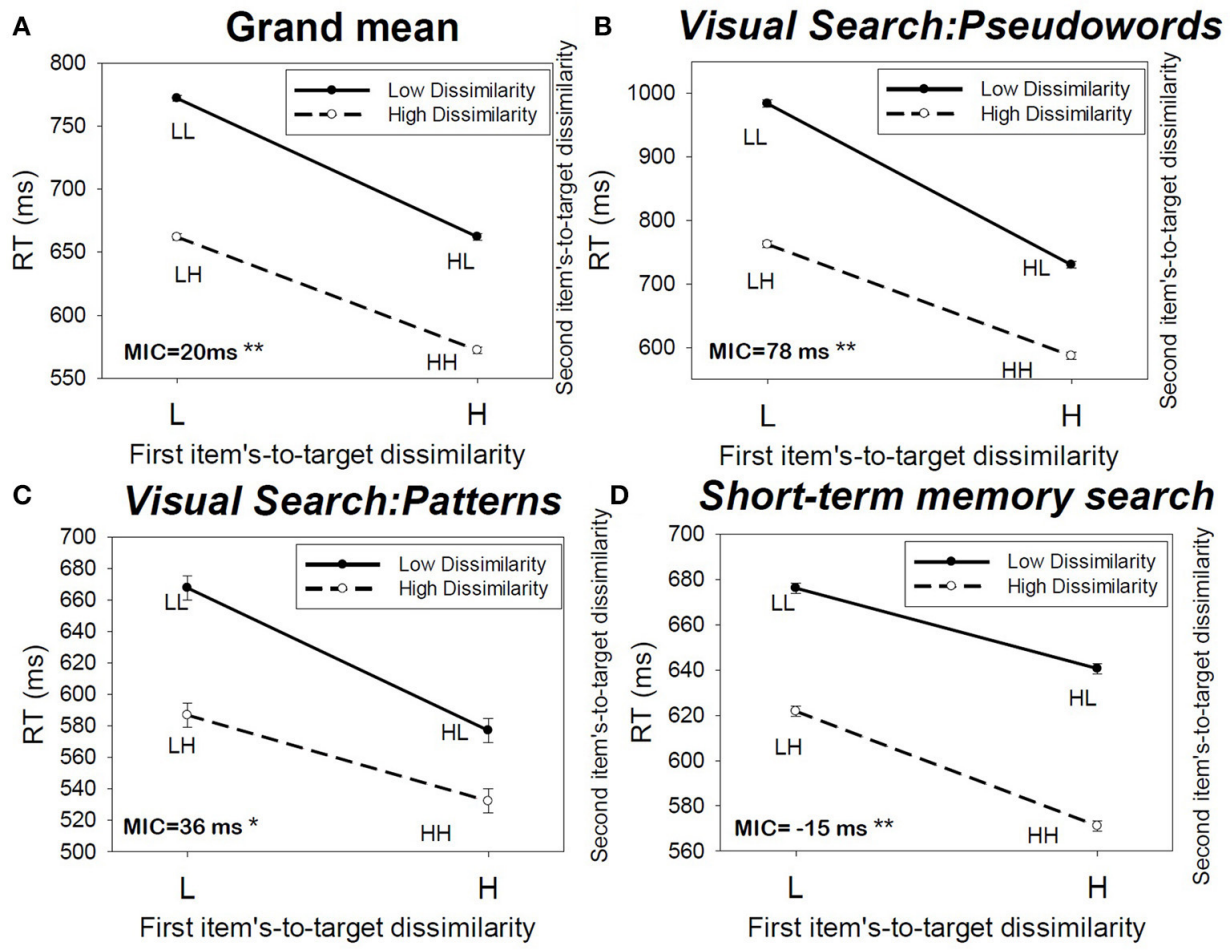
**(A)** Mean RT averaged across the subjects, and **(C,D)** the MIC test results for different experimental conditions.

First I analyzed the MIC results averaged across subjects and then across all experimental conditions (the visual and memory search conditions) to and obtained the grand mean MIC data (Figure 3A). Then, using ANOVA I tested the significance of the interaction between two factors. Each factor is defined as the item's item-to-target-dissimilarity (high, low), for one of the two positions in the search set position. The interaction test is used to provide a statistical significance finding for the MIC test. The interaction between the two factors was found to be significant *F*_(1, 23992)_ = 15.37, *p* < 0.01, η^2^ = 0.001. The observed MIC = 20 ms, indicating overadditivity (Figure 3, top left panel).

#### Conclusion: all subjects processing (26) was based on the coactive processing model

Next, I conducted the MIC test conditioned on the type of cognitive task used. I break down the overall mean RT results into three different experimental studies: the visual search task using pseudowords, visual search task using visual patterns, and short-term memory task. The results of MIC tests are presented in Table 2 (the rows “*Mean subjects,”* and also in Figures 3B–D).

#### Conclusion

The results indicated that when the MIC is calculated by averaging across all subjects the MIC test showed overadditivity (MIC > 0) in both of the visual search tasks, thus implying coactive processing (for 12 + 4 subjects). In a sharp contrast, the MIC indicated underadditivity (MIC < 0) in the short-term memory experiment, thus implying parallel processing, for all 10 subjects.

### Condition 2: individual subject data, regression analysis

The individual mean RT-dissimilarity functions are analyzed. The author conducted the linear regression analysis between mean RT and the number of item-to-target dissimilar items in a search set (0, 1, 2 items in a search set dissimilar to the target) for each individual subject across different experimental conditions (Table 4, left hand side).

**Table 4:** **Summarized linear regression results for different levels of subject analysis**.

	**Linear regression**				**Concavity/convexity test**			
	***R*^2^**	***F***	**Intercept (ms)**	**Slope (ms)**	***R*^2^ change**	**df2**	***F* change**	**Inference**
**GRAND MEAN**								
	1.00	299.4	769	−100	0.001	23993	15.38^**^	Coactive
**VISUAL SEARCH PSEUDOWORDS**								
*Mean subjects*	0.99	74.7	965	−196	0.005	7459	49.97^**^	Coactive
**Complexity**								
C = 1	0.94	16.4	625	−44	0.005	596	2.97^†^	Serial
C = 2	0.97	27.3	1077	−263	0.026	634	33.86^**^	Coactive
C = 3	1.00	399.2	1292	−361	0.002	632	4.33^*^	Coactive
C = 1	0.99	169.5	555	−44	0.002	592	1.20	Serial
C = 2	0.96	21.5	886	−177	0.032	631	40.64^**^	Coactive
C = 3	1.00	419.0	1140	−300	0.002	627	3.10^†^	Serial
C = 1	0.99	68.0	619	−44	0.001	591	0.86	Serial
C = 2	0.95	18.4	934	−218	0.042	633	59.21^**^	Coactive
C = 3	0.99	100.4	1173	−306	0.009	629	13.90^**^	Coactive
C = 1	0.98	49.2	675	−35	0.003	596	2.05	Serial
C = 2	0.97	27.6	1170	−214	0.026	634	32.57^**^	Coactive
C = 3	0.99	174.8	1431	−347	0.006	631	10.91^*^	Coactive
**VISUAL SEARCH PATTERNS**								
*Mean subjects*	0.97	36.0	663	−69	0.002	2347	5.36^*^	Coactive
**Complexity**								
C = 1	0.99	69.4	855	−116	0.003	588	2.08	Serial
C = 1	0.92	10.8	739	−67	0.008	577	4.69^*^	Coactive
C = 1	0.99	76.4	517	−44	0.003	585	2.05	Serial
C = 1	0.98	54.1	542	−47	0.006	588	4.41^*^	Coactive
**MEMORY SEARCH**								
*Mean Subjects*	0.99	152.8	679	−53	0.001	14181	10.93^**^	Parallel
**Interstimulus interval**								
SI = 700	1.00	222.6	608	−50	0.001	1376	0.81	Serial
ISI = 700	1.00	465.7	633	−33	0	1646	0.21	Serial
ISI = 700	0.94	14.4	605	−40	0.009	1203	11.27^**^	Parallel
ISI = 700	0.99	97.6	745	−42	0.001	1395	2.16	Serial
ISI = 700	1.00	1132.0	785	−97	0	1440	0.35	Serial
ISI = 2000	1.00	926.6	627	−60	0	1380	0.32	Serial
ISI = 2000	0.97	35.2	644	−37	0.002	1711	3.77^†^	Serial
ISI = 2000	0.94	16.6	620	−50	0.011	1202	14.64^**^	Parallel
ISI = 2000	0.96	23.7	753	−38	0.004	1388	5.57^*^	Parallel
ISI = 2000	0.99	68.7	767	−85	0.003	1413	4.07^*^	Parallel

** *p < 0.01*,

* *p < 0.05*,

† *p < 0.08. Each linear regression was conducted with 1 degree of freedom for the concavity/convexity test. The first dfs were 1 as stated, and the df2s are reported in the table*.

Using linear regression, the linear relationship accounts for a large percent of mean RT variability for most of the subjects (it ranged from 94 to 100% across all subjects, with the mean *R*^2^ = 98% and *SD* = 0.0282).

#### Conclusion 1

Extremely high *R*^2^-values of linear function fits among subjects implied a strict serial exhaustive process.

It is questionable whether the results would indicate significant curving of the mean data points, either of the convex or concave type. The standard way to test whether the data could be better explained by the linear or non-linear (polynomial of a second degree) model, is to conduct the regression analysis using the second-order polynomial regression function (quadratic). But in this study the use of quadratic regression is precluded as there are only three data points to be fitted. That is, there would be the same number of free parameters as the number of points, so the test for the significant *R*^2^ change from a linear to non-linear model would not be valid.

To provide the alternative test for curvature of the mean RT dissimilarity data the author conducted another regression analysis on the individual subject RT data this time by using all RTs not averaged across the dissimilarity conditions (0, 1, 2). Now the author compared whether the adding of a second order polynomial component could be used to significantly improve the goodness of fit (*R*^2^-value) (Table 4, right hand side).

#### Conclusions 2

The results of the regression analysis showed a significant curving of the individual subject data (Table 4, under Concavity/convexity test). The inferences about cognitive processes paralleled those of the MIC tests conducted on individual subjects' data (Table 2).

The only exception was the first subject whom was categorized now as a serial processor unlike in the MIC test in which this subject couldn't be classified in one of the three processing strategies.

### Condition 3: averaged subjects' data, regression analysis

First, I analyzed the data when averaged across subjects (individual data combined from the three experimental conditions). I conducted the linear regression analysis between mean RT and the number of item-to-target dissimilar items in a search set (0, 1, 2 items in a search set dissimilar to the target).

**Figure 4:**
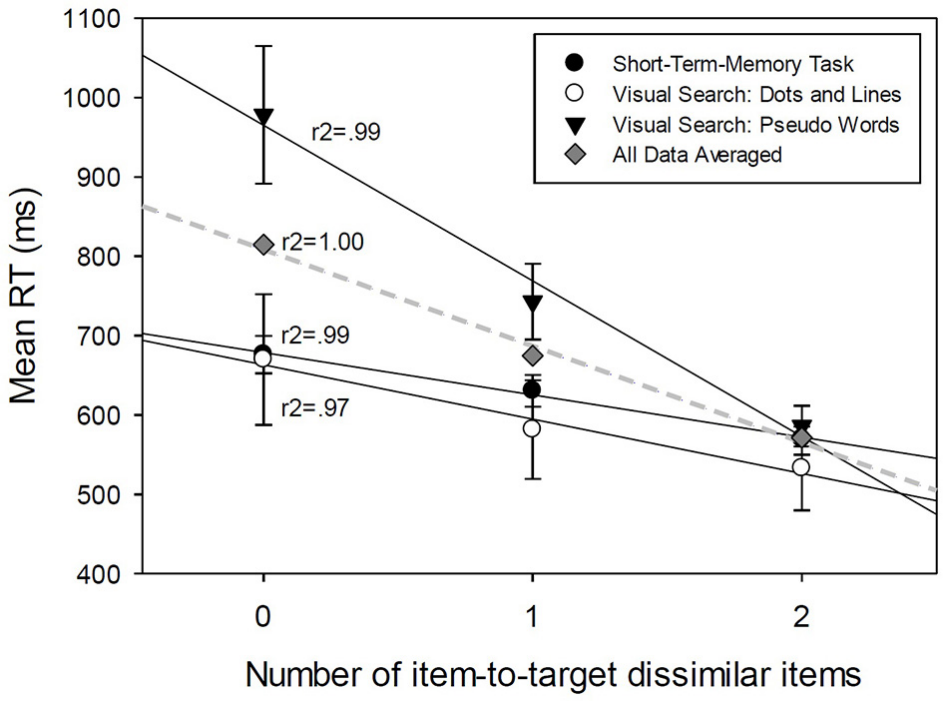
**Linear regression analyses between Mean RT averaged across the subjects and the number of item-to-target dissimilar items in a search set, for different experimental conditions**.

The significant proportion of explained variability indicates that the mean RT linearly decreases with increasing the number of items that are dissimilar to the target (see Figure 4, and Table 4 the first row *Grand Mean*). This relationship accounts for 100% of mean RT variability, *R*^2^ = 1 (Figure 4).

#### Conclusion 1

All subjects (26) processed the stimuli using the serial processing strategy. The rate of sequential processing per item is defined by the value of the regression function slope which was estimated from data to be 100 ms per item.

Second, I conducted the regression analysis on RT averaged across subjects but sorted by type of experimental condition. I break down the overall mean RTs into three different experimental studies: the visual search task using pseudowords, the visual search task using visual patterns, and the short-term memory task.

The results of the linear regression analysis between the mean RT and the number of dissimilar items are presented in Table 4 (the rows *Mean subjects*) and Figure 4. All three relationships accounted for between 97 and 99% of mean RT variability (0.97 ≤ *r*^2^ ≤ 0.99). The explained variability indicated that the mean RT linearly decreases with the number of items that are dissimilar to the target (Figure 4).

#### Conclusion 2

All subjects (26) used the serial cognitive processing strategy across different conditions. The rate of sequential processing per item was different for different experimental studies (see Figure 4) and varied between 196 ms per unit for pseudowords to 68 and 53 ms per unit for simple visual stimuli and STM search.

## General discussion

The main goal of the current paper was to explore the diagnostic accuracy of identifying the true underlying organization of cognitive processes in different experimental situations. The author discussed and analyzed two major concerns that could negatively impact the chances of achieving the main goals present in modern cognitive modeling trends.

The first concern deals with analyzing aggregated subjects data to infer the details associated with cognitive processes. Data aggregation across subjects has a long history of practice in the field. The main rationale is to use this powerful averaging tool to reduce random noise from observations and increase the power of diagnostic tests. The averaging tool rests on the conditional hypothesis that different subjects use the same cognitive operations. However, this hypothesis is rarely stated and substantiated. This is unfortunate, because when a researcher relaxes the conditional hypothesis that subjects use the same cognitive operations, surprising outcomes of averaging across subjects can occur. One of the most dramatic outcomes is inferring ghost cognitive processes. This error occurs when we average across two very different cognitive strategies. The resulting averaged data would support a strategy that may not exist and/or may not be theoretically feasible.

Research in the cognitive domain has over the years reached a critical view of the issue of individual differences in cognitive operations. It has become a pressing matter to address the issue of individual subject analysis. Scanning the current literature, the author found several such publications in the Journal of Psychological Review (Fific et al., 2010; Hills and Hertwig, 2012; Benjamin, 2013; Kellen et al., 2013b; Turner et al., 2013), the leading edge in theoretical advances relevant to the problem of averaging data across subjects.

The second concern deals with selection of the most appropriate research design to provide the best diagnostic performance in detecting cognitive processing details. A major trend in the cognitive domain relies on the principle that more complex designs make for better inferences. This is common practice in all areas of psychological research, which follows up on a recommendation for external generalizability. In that sense validation of a cognitive model should be based on the model's ability to generalize to as many as possible results and conditions as possible. In principle this is the right way to make scientific advances, especially in an area where it is not possible to precisely specify the minimal criteria for a research design complexity. For that reason the author introduced the SFT, which has been primarily designed to make inferences about underlying processing order (serial, parallel, coactive), stopping rule (terminating, exhaustive), and process dependency. The SFT approach proposes criteria for minimal research design complexity that can be used to construct the most effective diagnostic tools.

In this study the author reported the analysis of the effects of two possible ways inferences about cognitive processes can be jeopardized. The effect of the first jeopardy was measured by comparing the analysis of data averaged over the subjects to the analysis of individual subjects' data. The effect of the second jeopardy was measured by comparing the results of the analysis of the full factorial design (MIC) to the comparable FFD (linear regression on RT-difference function). More importantly the author cross combined the levels of jeopardies in a 2 × 2 analysis, leading to four different analysis conditions (Table 1). Condition zero served as a reference condition as it was the least influenced by both jeopardies. Table 3 shows the summary of inferences about the cognitive processes across the conditions.

Aggregating the data across subjects (Jeopardy 1) reduced the diagnostic accuracy of our inferences about cognitive process to about half (accuracy = 13/26). The analyses of the effect of subjects' data aggregation (Condition 1 and 0), showed not only omissions in detecting of some cognitive strategies, such as missing to detect 12 cases of serial processing, but also showed a number of false recognitions of parallel or coactive processing. Comparing the diversity of individual strategies revealed by the MIC test in Condition zero to the strategies inferred after the data aggregation shows an interesting finding. The resulting aggregated inferences are not necessarily affected by the most inferred individual cognitive processes. As shown in the memory search experiment, the individual MIC analyses indicated 6 serial and 4 parallel subjects (Table 2 bottom part—short term memory search). However, the inferences based on the aggregated values indicated parallel processing for all subjects (Table 2 the line “*mean subjects*” for short-term memory). This could happen as the aggregated MIC score accumulated the size of effects from the individual subjects' data. The individual MIC scores showed 7 negative values, of which only 4 reached significance and were inferred to occur in parallel (Table 2, bottom).

Collapsing across the full-factorial research design to create a less complex design (Jeopardy 2) showed very good diagnostic accuracy of cognitive processes. Using the FFD as an alternative to the full-factorial design led to 25/26 correct inferences (see summary in Table 3, Condition 2, the individual results in Table 4). The study can conclude that the shape of the RT-difference function can be used as a complement to the MIC test.

However, this comes with three caveats. First, using the FFD would be very ineffective if the data was aggregated over the subjects (as presented in Figure 4). The results of regression analysis on the data aggregated over the subjects showed impressive fits to linear functions and showed very high *R*^2^-values for each experiment. These results all point to the across subject uniform conclusion: serial processing (with low accuracy = 12/26). Second, even when the mean RT-difference functions are calculated for each separate subject (the Results Section, Condition 2, Conclusion 1) the curving of RT-difference functions may be difficult to detect using the conventional statistical test to reject the null hypothesis. To get the 25/26 correct detections, not only is the individual subjects analysis recommended but it is also recommended to use all data for each subject to test the curvature hypothesis (left-hand side Table 4, Concavity/convexity test). And the third and the most important caveat: using the FFD will very likely lead to increasing false alarm rates in detecting the known cognitive strategies, serial, parallel, or coactive. When scrutinized closely (Supplementary Material), the proposed FFD design shows good performance in inferring the correct cognitive strategy when all SFT conditional assumptions were met. However, if some of these assumption were not met, then FFD may not be able to detect that a violation occurred and will proceed to the incorrect inference. This is because FFD cannot test the mean RT ordering RT_LL_ > RT_LH_, RT_HL_ > RT_HH_, as the two LH and HL situations are aggregated. One such case is shown in Table 3 and also in Table 2, the first row with C = 1. The subjects' MIC RT data showed a violation of the mean RT ordering RT_LL_ > RT_LH_, RT_HL_ > RT_HH_ (Table 2, RT_LL_ = 619 ms, RT_LH_ = 564 ms, RT_HL_ = 623 ms, RT_HH_ = 530 ms) rendering the MIC test not valid for making inferences. The MIC test indicated that it is highly likely that some part of the conditional hypothesis was violated, thus preventing us from reaching a clear conclusion. However, when the FFD design is used the ordering of mean RTs allows for inferences (RT_LL_ = 619 ms, RT_LHandHL_ = (564 ms + 623 ms)/2 = 593 ms, RT_HH_ = 530 ms). The FFD design falsely inferred that this subject was a serial processor. In general the proposed FFD design is not an accurate test for the detection of “unknown” cognitive processes. The proof is shown in the corollary Supplementary Material.

Combining both jeopardies led to 12/26 correct inferences of serial processing (Table 3, Condition 3, see also Figure 4, “grand mean”). The linear regression analysis of RT-difference functions showed very high *R*^2^-values of linear functions across different experiments, leaving practically no room for curving, and detection of either parallel or coactive processing. Thus, the results did not infer any parallel or coactive strategies which constitute almost half of the individual result's analyses. The disappointingly low level of 46% correct inferences clearly warrants the use of better methods. In the relevant published work so far the author was able to find several studies that may be characterized as using the Condition 3 methods (for example, Lachmann and Geissler, 2002; Lachmann and van Leeuwen, 2004) and thus could be challenged for the validity of their inferences about cognitive processes.

The results of the current study lead to the following recommendations. To improve the diagnostic accuracy of cognitive process, it is advisable to avoid the jeopardies by both adopting the minimal research design criteria as proposed by SFT, and also by conducting individual subject analysis, rather than conducting the analysis on aggregated subject data. Both jeopardies have been recognized in the scientific community as having detrimental effects on inferences but infrequently taken care of.

A review of current research trends reveals a number of researchers who are ready to switch from the subject aggregating procedures, and instead consider using individual subject analysis, if they are not already en route to developing and using such methods (e.g., Myung et al., 2000; Brown and Heathcote, 2003; Estes and Maddox, 2005; Soto et al., 2014). The main challenge in using individual subject data is to provide an integral assessment of such data that can enable clear communication between researchers. This is the case when one has to report a variety of individual differences in a large data set. Another issue is the question of what the best statistical methodology is for analyzing data while allowing for individual assessment. Some researchers have suggested using hierarchical Bayesian statistical inference as a principle tool for hypotheses testing, as it allows for natural incorporation of individual difference as a part of statistical tests (e.g., Rouder and Lu, 2005; Lee, 2008; Liu and Smith, 2009; Bartlema et al., 2014).

In this paper the author recommends that the research community pay attention to recent methodological advances that allow for specification of criteria for the minimal complexity of research designs. The SFT proposes that (a) each cognitive process should be controlled by a separate experimental factor over the manipulated process saliency, and (b) The saliency levels of all factors should be combined in a full factorial design. The factor's saliency is a manipulation designed to *selectively influence* the speed of a certain cognitive process, so that the process is either speed up or slowed down (by provision of the selective influence). The minimal research design complexity is defined to be composed of 2^*n*^ experimental conditions. If your research design of exactly *n* number of processes has less than 2^*n*^ experimental conditions it is likely that the results of such a study will not be conclusive about the organization of the cognitive processes of interest. In that case, you may rather seek external generalizability, which will improve the likelihood of making correct inferences about the cognitive processes, though at an unknown rate.

### Conflict of interest statement

The author declares that the research was conducted in the absence of any commercial or financial relationships that could be construed as a potential conflict of interest.

## Footnotes

^1^In this study the terms cognitive strategies and cognitive operations are used interchangeably to refer to a set of mental processes organized in an identifiable mental network used in a specific task. In relevant literature these networks are also defined as mental architectures. In contrast, a cognitive process is subordinate term and indicates a single mental operation or a component of more complex cognitive system (mental architecture).

^2^Even this is not sufficient, e.g., every subject is exponential but their average appears to be from a different type of process (e.g., Brown and Heathcote, 2003).

^3^The current study doesn't evaluate model complexity as a quantitative criterion for model selection and falsification. The reasons are two-fold: (a) Current instantiation of SFT doesn't depend on model complexity to diagnose underlying cognitive models, it rather relies on recognition of qualitative patterns of RT and is completely non-parametric (for the parametric SFT approach see Fific et al., 2010). Nevertheless, one can argue that in the current paper the quantitative model comparison is possible as hypothesis testing is used to falsify certain classes of cognitive models. For example, in this paper the linear regression design is compared to the full factorial 2 × 2 ANOVA. (b) However, model selection is not necessary in this study: The linear regression model although a simpler model than the comparable full-factorial SFT design, makes logically incorrect inferences (as demonstrated in Supplementary Material). In such a case model complexity is a less important criterion to consider as one of the models is logically flawed.
