# Supplementary material for: Double jeopardy in inferring cognitive processes
Source: Front Psychol. 2014 Oct 21;5:1130. doi: 10.3389/fpsyg.2014.01130 (PMC4204447; doi:10.3389/fpsyg.2014.01130)
Supplement: Supplementary file 1 [file Presentation1.PDF]

Appendix A

Proposition: Response time (RT) additivity of a two-factor interaction test implies a strictly linear RT function of the fractional-factorial design that has aggregated mixed-level factorial conditions. Overadditivity implies a convex non-linear RT function, and underaditivity implies a concave non-linear RT function.

Proof: Assume a cognitive network of two processes: position one and position two. Suppose that the full- factorial design includes two factors (F_1 and F_2) with two levels (L,H). A factorial combination of these factors and their levels produces four experimental situations (LL,LH, HL, and HH). The first letter indicates the saliency of the first factor affecting the process at position one. The second letter indicates the saliency of the second factor affecting the process at position two. A researcher measures four mean response times (RTs) to complete two processes in an unknown cognitive network: RT_{LL}, RT_{LH}, RT_{HL}, and RT_{HH}.

Provided that the following conditions hold: (a) The processing rate for any position L is always slower than H, (b) The single factors each selectively influence only a single sub-process (position one and two), and (c) the independence between processes hold.

Under the conditions above, the mean response times in this simple cognitive network would produce the mean RT ordering of the four experimental situations $RT_{LL}>RT_{LH}$, $RT_{HL}> RT_{HH}$ (Townsend & Nozawa, 1995). Also, define the single mixed-level factorial situation as an aggregate between the LH and HL situations ($RT_{HL\&LH} =(RT_{HL} + RT_{LH})/2$), such that the full

factorial design is reduced to the fractional-fractional factorial with a one-dimensional factor with three levels (LL, LH&HL, HH).

Then the following is proposed:

MIC additivity → linear function(RT_{LL}, $RT_{HL\&LH}$, RT_{HH}) (1)

The left term of (1) could be written as the interaction test:

$RT_{LL} - RT_{HL} - RT_{LH} + RT_{HH} = 0$ (2)

The second term in (1) implies the following linear increment relationship:

$RT_{LL} – RT_{HL\&LH} = RT_{HL\&LH} - RT_{HH}$

$RT_{LL} – (RT_{HL} + RT_{LH})/2 = (RT_{HL} + RT_{LH})/2 - RT_{HH}$ (3)

By rearranging the terms in (3) we get the following expression:

$RT_{LL} - (RT_{HL} + RT_{LH})/2 - (RT_{HL} + RT_{LH})/2 + RT_{HH} = 0$

which is equal to (2)

$RT_{LL} - RT_{HL} - RT_{LH} + RT_{HH} = 0$

Thus MIC=0 implies the strictly linear RT function of the fractional factorial design defined above.

Under the same conditions, overadditivity of MIC>0 implies a convex non-linear RT function as:

$RT_{LL} - RT_{HL} - RT_{LH} + RT_{HH} > 0$

$RT_{LL} - RT_{HL} > RT_{LH} - RT_{HH}$,

This could also be represented as

$RT_{LL} – (RT_{HL} + RT_{LH})/2 > (RT_{HL} + RT_{LH})/2 - RT_{HH}$, and that

$RT_{LL} – RT_{HL\&LH} > RT_{HL\&LH} - RT_{HH}$

which is, when plotted, a convex nonlinear function.

And underadditivity (MIC<0) implies a convex non-linear function such that:

$RT_{LL} - RT_{HL} - RT_{LH} + RT_{HH} < 0$

$RT_{LL} - RT_{HL} < RT_{LH} - RT_{HH}$,

This could also be represented as

$RT_{LL} – (RT_{HL} + RT_{LH})/2 < (RT_{HL} + RT_{LH})/2 - RT_{HH,}$ and that

$RT_{LL} – RT_{HL\&LH} < RT_{HL\&LH} - RT_{HH}$

which, when plotted, is a concave nonlinear function.

The reverse of the proposal doesn’t hold in general. The shape of the above fractional-factorial RT function doesn’t imply the value of the MIC. It can only under the conditions specified above. However, these conditions (a, b, c) can only be tested under the full-factorial design. So the fractional-factorial design doesn’t imply the full factorial outcome.

Corollary: the fractional-factorial shape of the RT function (linear, convex, concave) can’t be used to verify whether the required conditions (a), (b) and (c) hold, as one can only observe the single mixed-level factorial situation LH&HL and two other situations HH and LL. These three situations are not sufficient to test the conditional relationship of the mean RT ordering:

$RT_{LL}>RT_{LH}$, $RT_{HL}> RT_{HH}$.

The ordering of

$RT_{LL} >RT_{HL\&LH}> RT_{HH}$

doesn’t imply the ordering of $RT_{LL}>RT_{LH}$, $RT_{HL}> RT_{HH}$,

as it is possible to construct the following situation in which

$RT_{LH}>RT_{LL}$, $RT_{HL}> RT_{HH}$, (4)

and that the following order holds $RT_{LL} >RT_{HL\&LH}> RT_{HH}$ (5)

This is because $RT_{HL\&LH}=(RT_{HL} + RT_{LH})/2$. One can find the appropriate values of RT_{HL} and RT_{LH} that can satisfy both the reverse ordering of the full-factorial design (4), and the ordering of the fractional-factorial design (5) (left to a reader).

Thus the fractional-factorial shape of the RT function provides the necessary but not sufficient evidence to infer the relationship between the four full-factorial conditions and thus can’t imply the corresponding MIC value.

In practice this means that the diagnostic fractional-factorial shape of the RT function can’t match the diagnostic accuracy of the of MIC test.
